# Recurrence quantification analysis of the effects of height-induced postural threat and arm movement restriction on balance performance in children and young adults

**DOI:** 10.1007/s00221-026-07428-1

**Published:** 2026-11-08

**Authors:** Johanna Lambrich, Anna M. Wissmann, Mathew W. Hill, Thomas Muehlbauer

**Affiliations:** 1 Division of Movement and Training Sciences/Biomechanics of Sport, University of Duisburg-Essen, Gladbecker Str. 182, 45141 Essen, Germany; 2 School of Psychology and Vision Sciences, University of Leicester, Leicester, UK

**Keywords:** Upright standing, Postural control, Nonlinear analysis, Upper-body strategy, Age

## Abstract

Nonlinear methods such as recurrence quantification analysis (RQA) have been used to provide complementary information into the effects of height-induced postural threat, however, without considering age- and task-related aspects. Thus, RQA was applied to investigate the effects of height-induced postural threat and arm movement restriction on balance performance in children compared to adults. In Experiment 1, 49 participants (26 children, 23 young adults) performed the tandem stance, while 48 participants (25 children, 23 young adults) conducted the unipedal stance with contralateral leg swing in Experiment 2. In both studies, standing at ground-level (no threat) and 80 cm above ground (threat) was performed with both free and restricted arm positions. RQA was conducted on center of pressure data from two previously published studies. In both experiments, group-by-threat interactions were detected, with adults demonstrating decreases in RQA parameters (Experiment 1: %DET AP, ENT AP, %LAM AP; Experiment 2: TT AP) when standing at height but children showing no adjustments. However, restriction of arms did not change the observed patterns. Overall, RQA provided information about the temporal organization of COP dynamics (more variable, less predictable, less complex, more random, less discontinuous, and shorter periods of invariant movement patterns due to height) that complemented the previously reported linear measures.

**Supplementary Information:**

The online version contains supplementary material available at https://doi.org/10.1007/s00221-026-07428-1.

## Introduction

Humans routinely encounter situations that challenge postural stability and increase the risk of balance loss (Harris et al. 2023). For example, standing or walking on elevated surfaces can potentially threaten balance control. In previous studies (Wissmann et al. 2025a, 2026), linear analysis methods of center of pressure (COP) trajectories were used to investigate the effects of height-induced postural threat and arm movement restriction on balance performance while standing. In young adults, standing above ground level compared to at ground level resulted in a decrease in COP amplitude and an increase in COP frequency, which is commonly interpreted as postural “stiffening” response (Adkin and Carpenter 2018; Carpenter et al. 2001; Hill et al. 2023, 2024; Huffman et al. 2009; Laufer et al. 2006). Moreover, the stiffening response was amplified when arm movements were restricted (Wissmann et al. 2026). In children, however, diverging responses were observed, i.e., standing at height compared to ground was associated with an increase in COP amplitude and a decrease in COP frequency (Hill et al. 2024; Omorczyk et al. 2021) or no changes (Wissmann et al. 2025a), which was interpreted as potentially maladaptive and ineffective postural adaptation strategy (Hill et al. 2024). Thus, children react differently than young adults in situations that threaten their balance. This is explained by ongoing developmental, maturation, and growth-related processes that influence the regulation of balance (Hirabayashi and Iwasaki 1995; Orendorz-Frączkowska and Kubacka 2019; Shumway-Cook and Woollacott 1985; Sinno et al. 2021). More precisely, (Hirabayashi and Iwasaki 1995) showed that proprioceptive function developed early, visual function followed and reached the adult level at the age of ≈ 15 years. The vestibular function developed later and showed a considerably lower level compared to adults even at the age of 15 years.

Despite these insights, conventional linear COP measures and nonlinear approaches quantify different properties of postural control. Linear measures typically characterize features such as amplitude, frequency, or velocity, whereas nonlinear approaches can additionally characterize temporal organization and recurrence structure. Consequently, linear analysis methods have increasingly been complemented by nonlinear approaches that can account for the non-stationary behavior of the human body to control posture. Some studies (Ellmers et al. 2021; Ellmers and Young 2018; Fischer et al. 2023; Hill et al. 2024) have used entropy-based measures, such as sample entropy, to quantify the irregularity of postural sway signals. However, sample entropy alone does not capture the temporal organization of fluctuations. Recurrence quantification analysis (RQA) goes beyond this by describing the temporal organization of postural dynamics. For example, it can reveal the degree of predictability and the presence of stable states, as well as how long the system remains in them. Notably, RQA metrics can supplement and refine results obtained from linear analysis methods, providing greater sensitivity in scenarios where linear effects are minimal or uncertain (Błażkiewicz et al. 2023; Riley et al. 1999). RQA is particularly advantageous as it requires no assumptions about data stationarity, distribution or length, and it remains robust to transients, outliers and noise (Labini et al. 2012; Riley et al. 1999). A recurrence plot is generated from a time series by reconstructing the system’s trajectory in higher-dimensional phase space using time-delay embedding. The positions of the trajectory at different time points are then compared in pairs. A recurrence is identified whenever the distance between two states falls below a predefined threshold, and this is marked as a point in the recurrence plot at the corresponding time coordinates (Eckmann et al. 1987; Weinberg et al. 2025). Several parameters can be derived from the recurrence plot: (a) recurrence rate (REC), which describes the proportion of all recurring states and indicates the degree of predictability in the system, (b) determinism (DET), which indicates the percentage of recurring points that form diagonal lines and indicates the degree of predictability in the system, (c) entropy (ENT), which quantifies the distribution of diagonal line lengths and indicates the complexity or irregularity of the movement patterns, c) average diagonal line length (LINE), which provides a measure of the average length of the diagonal lines and indicates the stability of predictable sequences, (d) laminarity (LAM), which describes the percentage of recurring points that form vertical lines and indicates phases with little change in state, and (e) trapping time (TT), which specifies the average length of vertical lines and indicates how long the system remains in a stable state (Błażkiewicz et al. 2023; Riley et al. 1999).

Height-induced postural threat has been associated with increased conscious control over movement (Hill et al. 2023, 2024), which would plausibly reduce predictability/regularity of postural sway independent of any change in sway magnitude. To date, only one study (Weinberg et al. 2025) has employed RQA in the context of height-induced postural threat. Investigating young healthy adults, (Weinberg et al. 2025) demonstrated that RQA metrics (i.e., DET, ENT, LINE) decreased when changing from the no-threat (i.e., quiet standing at ground level) to the threat (i.e., quiet standing above ground level) condition. This suggests less predictable and less automatic postural control, and more random movement patterns. While these findings provide the first evidence that height-induced postural threat can alter the temporal organization of sway dynamics in young adults, it remains unclear whether (i) similar effects are present in children which are characterized by an incomplete maturation of postural control mechanisms and (ii) the observed effects are influenced by restriction of arm movements.

## Experiment 1

The aim of the first experiment was to compare for the first time the effects of height-induced postural threat and different arm movement strategies between children and young adults using RQA. Although previous studies (Wissmann et al. 2025a, 2026) have investigated both factors (i.e., postural threat and arm movement restriction) in children versus young adults, they employed only linear analysis methods. In contrast, RQA has so far been used in one study (Weinberg et al. 2025), which examined the effect of height-induced postural threat but not restriction of arm movements in young adults only. Addressing these limitations, the present Experiment 1 applied RQA to compare children with young adults. Specifically, both age groups were asked to perform a static balance task (i.e., quiet tandem stance) both with free and restricted arm movements at ground level (no threat) and above ground level (threat). In previous studies—using linear analysis methods—height-induced postural threat led to different adjustments in children compared to young adults (Hill et al. 2024), which were further amplified by the restriction of arm movements (Wissmann et al. 2026). In another study using RQA (Weinberg et al. 2025), the authors reported significant decreases in the parameters DET, ENT, and LINE when standing on height compared to ground, indicating less predictable and less automatic postural control, and more random CoP patterns.

In sum, we expected that (1) RQA parameters would decrease from no threat to threat conditions, reflecting changes toward less predictable recurrence structures; (2) restriction of arm movements would further lower the RQA parameters, because removing compensatory degrees of freedom may force reliance on more effortful, less automatic control strategies; but (3) these results observed in young adults would differ from those in children due to their immature postural control.

### Materials and methods

This dataset was derived from an earlier publication (Wissmann et al. 2025a) that used linear analysis methods to compare the impact of height-induced postural threat and restricted arm movements between children and young adults using a static balance task.

#### Participants

Twenty-six children (mean age = 9.8 ± 0.6 years, 13 males) and 23 young adults (mean age = 24.7 ± 4.0 years, 12 males) participated in this experiment. All participants had no prior experience with experimental conditions and were not aware of the specific purpose of this study. They reported no musculoskeletal dysfunction, neurological impairment, or orthopedic disorder. Prior to the experiment, written informed consent was obtained from all participants and their legal guardians if required. The experiment was conducted with approval (EA-PSY9/24/25032024) of the Human Ethics Committee at the University of Duisburg-Essen, Faculty of Educational Sciences.

#### Experimental procedure

**Fig. 1:**
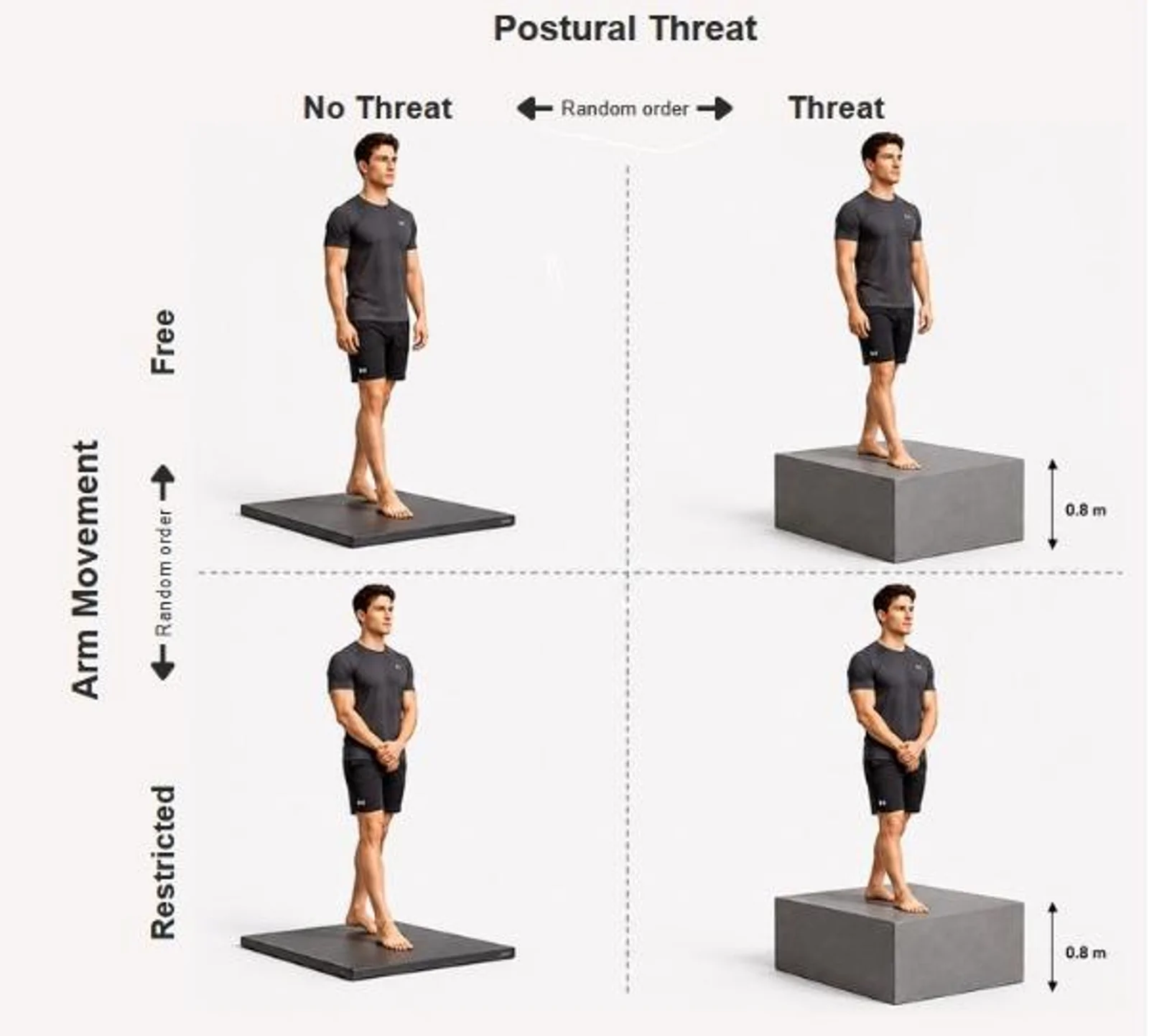
Experimental procedure for Experiment 1

Participants were asked to stand barefoot in tandem stance (i.e., right foot positioned in front of the left foot) on a force plate (AMTI, AccuGait, Watertown, MA, USA; dimensions: 50 × 50 × 5 cm). Each participant completed a 30-s practice trial followed by a 60-s measurement trial under different postural threat and arm movement conditions applied in a random order (Fig. 1): standing at ground level (no threat) and 80 cm above ground level (threat) accessible via a staircase with free (i.e., arms moved freely) and restricted (i.e., hands clasped in front of the body at waist level) arm movements. All trials were completed without using a safety harness and participants were instructed to stand quietly while gazing at a black cross (adjusted to the individuals eye level) three meters away from the force plate.

#### Measurements

Ground reaction forces were sampled at 1000 Hz (Netforce, AMTI, Watertown, MA, USA) and low-pass filtered (cut-off frequency: 5 Hz) offline using a bidirectional, second-order Butterworth filter.

#### Data analysis

Prior to the RQA, the COP time series were resampled to 100 Hz. RQA was performed using the corresponding toolbox for MATLAB (Ouyang 2022), following the methodological recommendations outlined in the systematic review by Błażkiewicz et al. (2023). To ensure methodological consistency and enable direct comparison with the findings of Weinberg et al. (2025), the embedding dimension (m = 3) and time delay (τ = 6, i.e., 60ms) were adopted accordingly. Distances between embedded vectors were computed with the Euclidean norm.

Recurrence plots were constructed from the COP data. All analyses were performed separately for the anteroposterior (AP) and mediolateral (ML) sway directions. In accordance with the procedure described by Weinberg et al. (2025) the following RQA parameters were determined to ensure comparability across participants and conditions: %REC (i.e., percentage of recurrent points that can range from 0% [no recurrent points] to 100% [all points are recurrent] whereas a threshold of 5% ± 0.5% was applied (Riley et al. 1999; Webber and Zbilut 2005); %DET (i.e., percentage of recurrent points forming diagonal lines of at least two points, excluding the line of identity), with a lower value indicating a less predictable, less deterministic, and more random CoP movements (van den Hoorn et al. 2020); ENT (i.e., complexity of the diagonal line length distribution, computed as the Shannon entropy of the length of diagonal lines of at least two points, with probabilities normalized over all diagonal lines as implemented in the toolbox), with a lower value indicating less automatic and more conscious postural control (Bonnette et al. 2020; Donker et al. 2007); LINE (i.e., average length of all diagonal lines of at least two points), with a lower value indicating more random CoP patterns (van den Hoorn et al. 2020). Further and in accordance with Błażkiewicz and colleagues (2023), the following RQA parameter were additionally calculated: %LAM (i.e., percentage of recurrent points forming vertical lines), with a lower value indicating less discontinuous CoP movements with fewer periods of minimal CoP fluctuations (Bonnette et al. 2020; van den Hoorn et al. 2020); TT (i.e., mean duration that the system remains in a laminar state, computed as the mean length of these vertical lines), with a lower value indicating shorter periods of CoP fluctuations (Bonnette et al. 2020). Standing at ground level with free arms served as the baseline condition (Table S1, supplementary material). In this condition, an individual threshold was determined during the baseline condition as a percentage of the standard deviation of the baseline COP signal and subsequently applied unchanged to all remaining conditions for that participant. Consequently, the RQA measures are invariant to the overall magnitude of the COP signal. They may nevertheless covary with sway amplitude across conditions if changes in amplitude are accompanied by changes in the temporal structure of sway. No Theiler window was applied. Because the embedding parameters were not derived from the present data, we estimated them in baseline trials (first minimum of the average mutual information, false nearest neighbor criterion of < 1% (Kennel et al. 1992). In addition, we conducted a sensitivity analysis (Marwan 2011) in which the RQA, including the threshold calibration, and all ANOVAs were recomputed for seven alternative settings, each differing one parameter from the main analysis: $\:\tau\:\:=\:40\:ms,\:\tau\:=\:80\:ms$; $\:\tau\:$ based on the average mutual information (Experiment 1: 640 ms, Experiment 2: 400 ms), m = 4, a Theiler window of 120 ms, and minimum line length of 3 and 10 samples (Table S2 and S3, supplementary material). In addition, a multidimensional RQA (Wallot et al. 2016) was performed on the two-dimensional COP trajectory: AP and ML were z-standardized per trial, embedded with m = 3 and $\:\tau\:$ = 60 ms and combined into one state vector; all further steps were identical to the main analysis.

#### Statistical analyses

All statistical analyses were conducted using JASP version 0.19.2 (Amsterdam, The Netherlands) for analyses of variance and Python (version 3.14) for the repeated-measures correlations. Prior to performing parametric tests, assumptions of normality (i.e., Shapiro–Wilk test) and homogeneity of variances or sphericity (Mauchly’s test) were checked and met. Afterwards, a series of mixed-model analyses of variance (ANOVA) were applied to evaluate the between-subject effect of age group (two levels: children vs. young adults) and the within-subject effects of postural threat (two levels: no threat vs. threat) and arm movement condition (two levels: free vs. restricted). When a significant interaction effect emerged, Bonferroni-corrected *post hoc* tests were carried out to identify the specific pairwise differences. For the ANOVAs, effect size was quantified using partial eta squared (${{\eta\:}}_{{p}}^{2}$) and interpreted as small (0.02 ≤ ${{\eta\:}}_{{p}}^{2}$ ≤ 0.12), medium (0.13 ≤ ${{\eta\:}}_{{p}}^{2}$ ≤ 0.25), or large (${{\eta\:}}_{{p}}^{2}$ ≥ 0.26). For the pairwise comparisons, Cohen’s *d* was calculated (Cohen 1988) and classified as trivial (0 ≤ *d* ≤ 0.19), small (0.20 ≤ *d* ≤ 0.49), moderate (0.50 ≤ *d* ≤ 0.79), or large (*d* ≥ 0.80). The alpha level for statistical significance was set a priori at *p* < 0.05 for all tests. To examine whether RQA parameters provide information beyond the previously reported linear measures (Wissmann et al. 2025a, 2026), repeated-measures correlation (Bakdash and Marusich 2017) were computed. *P*-values were adjusted using Benjamin-Hochberg false discovery rate (Benjamini and Hochberg 1995).

### Results

Table 1 presents the group means ± standard deviations and Table 2 shows the ANOVA results for all assessed variables.

#### Determinism, entropy, and diagonal line length

All ANOVAs conducted on %DET, ENT, and LINE revealed significant main effects of group (*p* < 0.001) and threat (*p* < 0.013) but not of arm (Table 2). Further, there were significant group × threat interactions for %DET AP (*p* < 0.001, $\:{\eta\:}_{p}^{2} = 0.27$) and ENT AP (*p* = 0.021, $\:{\eta\:}_{p}^{2}$ = 0.11). For %DET AP, *post-hoc* analysis identified a significant decrease from standing at to above ground level (irrespective of arm movement condition) in young adults (*t* = 6.780, *p* < 0.001, *d* = 0.72) but not in children (*t* = 1.135, *p* = 1.000, *d* = 0.11). Regarding, ENT AP, *post-hoc* analysis revealed a significant decrease from standing on ground to height (irrespective of arm movement condition) in young adults (*t* = 4.474, *p* < 0.001, *d* = 0.73) but again not in children (*t* = 1.277, *p* = 1.000, *d* = 0.20).

#### Laminarity and trapping time

All ANOVAs performed on %LAM and TT yielded significant main effects of group (*p* < 0.001) and threat (*p* < 0.05) but not of arm (Table 2). Additionally, there was a significant group × threat interaction for %LAM AP (*p* < 0.001, $\:{\eta\:}_{p}^{2}$ = 0.23). *Post-hoc* analyses indicated a significant decrease from standing at to above ground level (irrespective of arm movement condition) in young adults (*t* = 6.121, *p* < 0.001, *d* = 0.86) but not in children (*t* = 1.086, *p* = 1.00, *d* = 0.14).

Sensitivity analyses confirmed all main effects of group and all group x threat interactions under all parameter settings (Table S2, supplementary material). Two-dimensional analysis showed main effects of group (*p* < 0.001) and threat (*p* ≤ .004) but no group x threat interactions (*p* ≥ 0.113) (Table S4, supplementary material).

#### Associations between linear (COP metrics) and non-linear (RQA metrics) measures

RQA parameters were positively associated with RMS (r_rm = 0.27 to 0.82) and negatively with sample entropy (r_rm =-0.47 to − 0.83) of the same sway direction (Figure S1, supplementary material).

**Table 1:** Group mean values ± standard deviations for all assessed variables by age group (children vs. young adults), threat conditions (no threat vs. threat), and arm conditions (free vs. restricted)

Parameter	Arm condition	Children (*n* = 26)		Young adults (*n* = 23)	
		No threat	Threat	No threat	Threat
REC AP (%)	Free	5.05 ± 0.26	5.20 ± 2.85	4.90 ± 0.29	3.67 ± 1.50
	Restricted	5.18 ± 1.61	4.83 ± 1.99	4.32 ± 2.25	3.70 ± 1.75
REC ML (%)	Free	4.99 ± 0.25	4.47 ± 1.05	5.11 ± 0.25	4.78 ± 1.13
	Restricted	5.07 ± 1.38	4.26 ± 0.94	5.09 ± 1.48	4.95 ± 1.83
DET AP (%)	Free	99.92 ± 0.02	99.91 ± 0.05	99.88 ± 0.06	99.77 ± 0.15
	Restricted	99.92 ± 0.03	99.91 ± 0.04	99.81 ± 0.15	99.79 ± 0.12
DET ML (%)	Free	99.91 ± 0.01	99.90 ± 0.02	99.86 ± 0.04	99.81 ± 0.09
	Restricted	99.91 ± 0.04	99.89 ± 0.03	99.85 ± 0.07	99.82 ± 0.11
ENT AP (nat)	Free	3.82 ± 0.14	3.80 ± 0.28	3.62 ± 0.23	3.37 ± 0.25
	Restricted	3.84 ± 0.22	3.77 ± 0.27	3.49 ± 0.26	3.39 ± 0.25
ENT ML (nat)	Free	3.73 ± 0.05	3.66 ± 0.11	3.48 ± 0.14	3.40 ± 0.18
	Restricted	3.74 ± 0.16	3.64 ± 0.10	3.47 ± 0.19	3.41 ± 0.22
LINE AP (points)	Free	20.51 ± 3.15	20.62 ± 5.61	17.29 ± 3.71	13.89 ± 2.98
	Restricted	21.39 ± 4.69	20.18 ± 5.38	15.49 ± 3.19	14.22 ± 2.95
LINE ML (points)	Free	18.67 ± 0.97	17.57 ± 1.74	14.82 ± 1.90	13.96 ± 2.15
	Restricted	19.12 ± 3.52	17.29 ± 1.42	14.90 ± 2.60	14.15 ± 2.53
LAM AP (%)	Free	99.96 ± 0.02	99.94 ± 0.05	99.93 ± 0.04	99.81 ± 0.16
	Restricted	99.95 ± 0.04	99.94 ± 0.05	99.86 ± 0.14	99.81 ± 0.16
LAM ML (%)	Free	99.95 ± 0.01	99.92 ± 0.04	99.90 ± 0.04	99.85 ± 0.09
	Restricted	99.94 ± 0.03	99.92 ± 0.04	99.88 ± 0.07	99.84 ± 0.14
TT AP (points)	Free	23.02 ± 5.86	23.15 ± 10.11	18.46 ± 5.50	13.01 ± 4.15
	Restricted	24.46 ± 8.99	22.26 ± 9.94	15.28 ± 4.52	13.25 ± 4.23
TT ML (points)	Free	18.92 ± 2.50	16.45 ± 3.76	14.01 ± 2.18	12.69 ± 2.63
	Restricted	19.40 ± 6.57	15.83 ± 2.95	14.06 ± 3.67	12.95 ± 3.38

AP, anteroposterior; DET, determinism; ENT, entropy; ML, mediolateral; LAM, laminarity; LINE, line length; nat, natural units of information; REC, recurrence rate (retained as a descriptive check but not as a primary inferential outcome); TT, trapping time

**Table 2:** Main and interaction effects of the repeated measures ANOVA for all assessed variables

Parameter	Group (children vs. young adults)	Threat (no threat vs. threat)	Arm (free vs. restricted)	Threat × Arm	Group × Threat	Group × Arm	Group × Threat × Arm
DET AP (%)	***F*** **= 28.245** ***p (η***_**p**_^**2**^ **)< 0.001 (0.38)**	***F*** **= 32.682** ***p (η***_**p**_^**2**^ **) < 0.001 (0.41)**	*F* = 1.651 *p (η*_p_^2^) = 0.205 (0.03)	*F* = 3.168 *p (η*_p_^2^) = 0.082 (0.06)	***F*** **= 17.316** ***p (η***_**p**_^**2**^ **)< 0.001 (0.27)**	*F* = 1.494 *p (η*_p_^2^) = 0.228 (0.03)	*F* = 2.283 *p (η*_p_^2^) = 0.137 (0.05)
DET ML (%)	***F*** **= 39.237** ***p (η***_**p**_^**2**^ **) < 0.001 (0.46)**	***F*** **= 6.677** ***p (η***_**p**_^**2**^ **) = 0.013 (0.12)**	*F* = 0.365 *p (η*_p_^2^) = 0.549 (0.01)	*F* = 0.066 *p (η*_p_^2^) = 0.798 (0.00)	*F* = 1.291 *p (η*_p_^2^) = 0.262 (0.03)	*F* = 0.025 *p (η*_p_^2^) = 0.876 (0.00)	*F* = 1.373 *p (η*_p_^2^) = 0.247 (0.03)
ENT AP (nat)	***F =*** **56.789** ***p (η***_**p**_^**2**^ **) < 0.001 (0.55)**	***F*** **= 17.089** ***p (η***_**p**_^**2**^ **) < 0.001 (0.27)**	*F* = 0.794 *p (η*_p_^2^) = 0.377 (0.02)	*F* = 0.742 *p (η*_p_^2^) = 0.393 (0.02)	***F*** **= 5.685** ***p (η***_**p**_^**2**^**) = 0.021 (0.11**)	*F* = 0.793 *p (η*_p_^2^) = 0.378 (0.02)	*F* = 2.616 *p (η*_p_^2^) = 0.112 (0.05)
ENT ML (nat)	***F =*** **67.767** ***p (η***_**p**_^**2**^ **) < 0.001 (0.59)**	***F*** **= 12.491** ***p (η***_**p**_^**2**^ **) < 0.001 (0.21)**	*F* = 0.002 *p (η*_p_^2^) = 0.963 (0.00)	*F* = 0.110 *p (η*_p_^2^) = 0.741 (0.00)	*F* = 0.139 *p (η*_p_^2^) = 0.711 (0.00)	*F* = 0.047 *p (η*_p_^2^) = 0.830 (0.00)	*F* = 0.649 *p (η*_p_^2^) = 0.424 (0.01)
LINE AP (points)	***F*** **= 55.833** ***p (η***_**p**_^**2**^**) < 0.001 (0.54**)	***F*** **= 7.047** ***p (η***_**p**_^**2**^ **) = 0.011 (0.13)**	*F* = 0.262 *p (η*_p_^2^) = 0.611 (0.01)	*F* = 0.125 *p (η*_p_^2^) = 0.725 (0.00)	*F* = 2.717 *p (η*_p_^2^) = 0.106 (0.06)	*F* = 0.910 *p (η*_p_^2^) = 0.345 (0.02)	*F* = 2.291 *p (η*_p_^2^) = 0.137 (0.05)
LINE ML (points)	***F*** **= 68.678** ***p (η***_**p**_^**2**^**) < 0.001 (0.59**)	***F*** **= 13.121** ***p (η***_**p**_^**2**^ **) < 0.001 (0.22)**	*F* = 0.224 *p (η*_p_^2^) = 0.639 (0.01)	*F* = 0.486 *p (η*_p_^2^) = 0.489 (0.01)	*F* = 1.109 *p (η*_p_^2^) = 0.298 (0.02)	*F* = 0.012 *p (η*_p_^2^) = 0.912 (0.00)	*F* = 0.897 *p (η*_p_^2^) = 0.348 (0.02)
LAM AP (%)	***F =*** **24.320** ***p (η***_**p**_^**2**^ **) < 0.001 (0.34)**	***F*** **= 27.072** ***p (η***_**p**_^**2**^ **) < 0.001(0.37)**	*F* = 2.356 *p (η*_p_^2^) = 0.132 (0.05)	*F* = 2.056 *p (η*_p_^2^) = 0.158 (0.04)	***F*** **= 13.798** ***p (η***_**p**_^**2)**^ **< 0.001(0.23)**	*F* = 1.307 *p (η*_p_^2^) = 0.259 (0.03)	*F* = 1.357 *p (η*_p_^2^) = 0.250 (0.03)
LAM ML (%)	***F =*** **28.479** ***p (η***_**p**_^**2**^ **) < 0.001 (0.38)**	***F*** **= 8.406** ***p (η***_**p**_^**2**^ **) = 0.006 (0.15)**	*F* = 2.570 *p (η*_p_^2^) = 0.116 (0.05)	*F* = 0.084 *p (η*_p_^2^) = 0.774 (0.00)	*F* = 0.615 *p (η*_p_^2^) = 0.437 (0.01)	*F* = 0.378 *p (η*_p_^2^) = 0.542 (0.01)	*F* = 0.076 *p (η*_p_^2^) = 0.784 (0.00)
TT AP (points)	***F*** **= 45.433** ***p (η***_**p**_^**2**^**) < 0.001 (0.49**)	***F*** **= 6.107** ***p (η***_**p**_^**2**^ **) = 0.017 (0.12)**	*F* = 0.423 *p (η*_p_^2^) = 0.519 (0.01)	*F* = 0.076 *p (η*_p_^2^) = 0.784 (0.00)	*F* = 1.963 *p (η*_p_^2^) = 0.168 (0.04)	*F* = 0.906 *p (η*_p_^2^) = 0.346 (0.02)	*F* = 2.044 *p (η*_p_^2^) = 0.159 (0.04)
TT ML (points)	***F*** **= 40.058** ***p (η***_**p**_^**2**^**) < 0.001 (0.46**)	***F*** **= 14.752** ***p (η***_**p**_^**2**^ **) < 0.001(0.24)**	*F* = 0.008 *p (η*_p_^2^) = 0.929 (0.00)	*F* = 0.281 *p (η*_p_^2^) = 0.599 (0.01)	*F* = 2.692 *p (η*_p_^2^) = 0.108 (0.05)	*F* = 0.062 *p (η*_p_^2^) = 0.804 (0.00)	*F* = 0.605 *p (η*_p_^2^) = 0.441 (0.01)

0.02 ≤ *η*_p_^2^ ≤ 0.12 indicates small, 0.13 ≤ *η*_p_^2^ ≤ 0.25 indicates medium, and *η*_p_^2^ ≥ 0.26 indicates large effects. Bold values indicate statistically significant differences (*p* < 0.05). AP, anteroposterior; DET, determinism; ENT, entropy; ML, mediolateral; LAM, laminarity; LINE, line length; nat, natural units of information; TT, trapping time

#### Discussion

As indicated by the main effect of postural threat, Experiment 1 provided compelling evidence that standing at height compared to ground led to significant decreases in all RQA parameters which are consistent with our first hypothesis and the findings of (Weinberg et al. 2025). The authors reported significant reductions in RQA metrics (i.e., %DET, ENT, LINE) when standing above compared to at ground level. Specifically, decreases in these RQA parameters indicate a lower predictability and reduced complexity in postural control, along with more random COP movements (Bonnette et al. 2020; Donker et al. 2007; van den Hoorn et al. 2020) and offer added value compared with conventional analyses (Wissmann et al. 2025a). Wissmann and colleagues(Wissmann et al. 2025b) also reported significant changes in COP frequency and velocity when standing above compared to on ground level (Table S5, supplementary material). Furthermore, we analyzed additional RQA parameters such as %LAM and TT for the first time and observed significant decreases. This is indicative of less discontinuous CoP movements and shorter periods of CoP fluctuations (Błażkiewicz et al. 2023; Bonnette et al. 2020; van den Hoorn et al. 2020).

Contrary to our assumption stating that restricted arm movements will further lower the RQA parameters, no significant threat-by-arm interactions were observed, meaning that the second hypothesis was not confirmed. A possible explanation for this finding is that the assumed negative effect of restricted arm movements on postural control may have been masked by a strong influence of height-induced postural threat. Furthermore, the quiet tandem stance presents a rather simple static balance task and restriction of arm movements when standing at height has no additional impact on postural control. Therefore, future work (see Experiment 2) should examine whether restriction of arm movement amplifies threat-induced decreases in RQA parameters when using a potentially more demanding semi-static balance task (e.g., unipedal stance with contralateral leg swing).

Consistent with our third hypothesis and previous research using linear analysis methods (Hill et al. 2024; Wissmann et al. 2025a) (Table S5, supplementary material), the detected significant group-by-threat interactions indicate differences between children and young adults when standing at height compared to ground level. Precisely, *post-hoc* analyses revealed decreases from standing on ground to height in RQA parameters (i.e., %DET AP, ENT AP, %LAM AP) in young adults but not in children. This finding suggests that, unlike children, young adults were able to adapt their postural control to balance-threatening situations. The absence of such a response in children could be due to an incomplete maturation of the underlying mechanisms (Shumway-Cook and Woollacott 1985). For example, using the Sensory Organization Test, (Sinno et al. 2021) examined differences in sensory contributions to postural control between children aged 5–17 years and young adults aged 20–25 years. Their findings indicated that proprioceptive function matures relatively early, reaching levels comparable to those of young adults by approximately 5–8 years of age. Visual function develops subsequently and appears to reach full maturity around 12–14 years, whereas vestibular function shows the latest maturation, achieving adult-like characteristics between 15 and 17 years of age.

The observed reductions in RQA parameters were limited to the AP sway direction. This finding can be explained by the fact that the platform edge was located in front of the participants, and previous studies (Cleworth et al. 2012; Zaback et al. 2019) have shown a shift in COP displacements in the AP direction (i.e., away from the platform edge) rather than in the ML direction.

## Experiment 2

The effect of restricted arm movements while standing at height did not emerge in Experiment 1. One possible explanation is that the static balance task used in Experiment 1 may have placed limited additional demands on postural control when arm movements were restricted. Consequently, Experiment 2 used a semi-static task involving continuous contralateral leg swing, which was expected to impose different and potentially greater coordination demands than quiet tandem stance. Specifically, the participants were instructed to perform a continuous contralateral leg swing during unipedal stance. The other two factors—postural threat and age group—were not altered. Previous studies using linear analysis reported that children showed significant adjustments in their motor responses during difficult tasks (Wissmann et al. 2026) that were not evident during easy tasks (Wissmann et al. 2025a). Therefore, the purpose of the second experiment was to replicate Experiment 1 but using a more difficult semi-static balance task. We hypothesized that (1) RQA parameters would decline from no threat to threat conditions, (2) restricted arm movements would further lower the RQA parameters, but (3) these changes detected for young adults would differ from those observed in children.

### Materials and methods

This dataset originates from a previous publication (Wissmann et al. 2026), in which linear analysis methods were used to examine differences in the effect of height-induced postural threat and arm moment restriction between children and young adults using a semi-static balance task.

#### Participants

The study sample comprised 25 children (mean age: 11.7 ± 0.5 years, 8 males) and 23 young adults (mean age: 21.8 ± 3.4 years, 14 males). None of the participants had prior experience with experimental conditions and were aware of the specific purpose of this study. They reported were free of any musculoskeletal dysfunction, neurological impairment, and orthopedic disorder. Before the study, they and their legal guardians provided written informed consent. The study protocol was approved by the Human Ethics Committee of the Faculty of Educational Sciences, University of Duisburg-Essen (approval number: EA-PSY9/24/25032024).

#### Experimental procedure

**Fig. 2:**
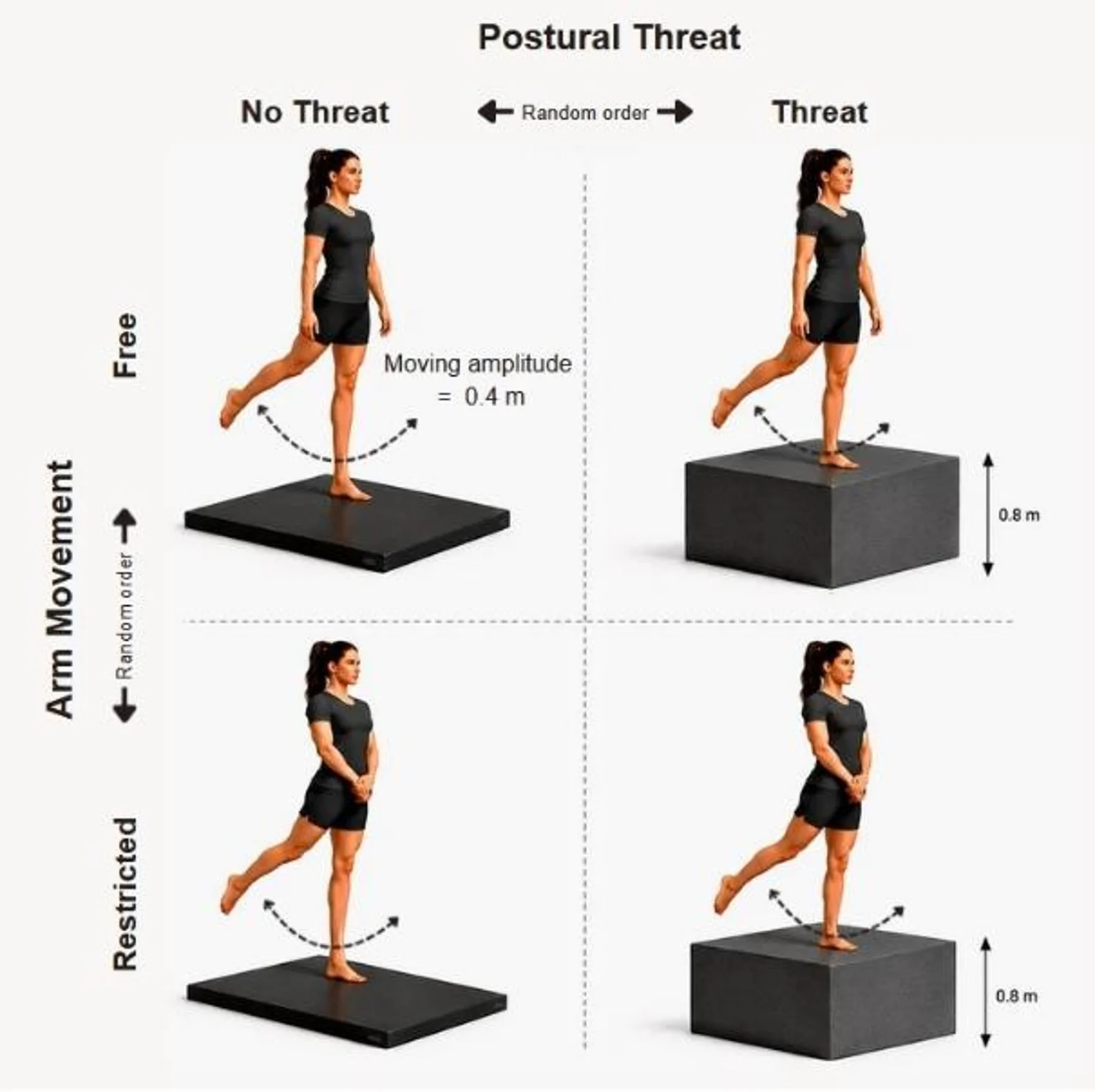
Experimental procedure for Experiment 2

The experimental procedure was identical to that used in Experiment 1 except that a semi-static task was used. Precisely, participants were instructed to perform rhythmic forward–backward swings of their dominant leg at a metronome-paced frequency of 2 Hz (Fig. 2). A movement amplitude of 40 cm was prescribed, marked by indicators placed 20 cm anterior and 20 cm posterior to the participant on the platform. Movement amplitude and frequency were visually monitored by an experimenter to ensure adherence to the protocol.

#### Measurements

Measurements were performed in the same manner as in Experiment 1.

#### Data analysis

Data analysis was identical to that applied in Experiment 1. The condition involving standing at ground level with free arm movements was designated as the baseline (Table S6, supplementary material), and the parameters derived from this condition were subsequently employed in the RQAs for the other experimental conditions.

#### Statistical analyses

Statistical analyses were identical to that conducted in Experiment 1.

### Results

Table 3 displays the group means ± standard deviations and Table 4 presents the ANOVA results for all assessed variables.

#### Determinism, entropy, and diagonal line length

All ANOVAs conducted on %DET, ENT, and LINE showed significant main effects of group (*p* < 0.001) but not of arm (Table 4). Further, the main effect of threat (*p* < 0.05) was significant for all measures, except one (i.e., LINE AP and LINE ML). In addition, there were threat × arm interactions for %DET ML (*p* = 0.049, ${{\eta\:}}_{{p}}^{2} = 0.08$), ENT ML (*p* = 0.024, ${{\eta\:}}_{{p}}^{2} = 0.11$), and LINE ML (*p* = 0.038, ${{\eta\:}}_{{p}}^{2} = 0.09$). For %DET ML, *post-hoc* analysis identified a significant decrease when standing above ground level (irrespective of age group) with free (*t* = 2.913, *p* = 0.033, *d* = 0.53) but not with restricted (*t* = 0.003, *p* = 1.0, *d* = 0.00) arm movements. Regarding ENT ML, *post-hoc* analysis revealed a significant decrease when standing at height (irrespective of age group) with free (*t* = 2.998, *p* = 0.026, *d* = 0.56) but again not with restricted arm (*t* = − 0.198, *p* = 1.000, *d* = 0.03) arm movements. In terms of LINE ML, *post-hoc* analysis showed a significant decrease in LINE ML when standing above ground level (irrespective of age group) with free (*t* = 2.983, *p* = 0.027, *d* = 0.46) but again not with restricted (*t* = − 0.466, *p* = 1.000, *d* = 0.10) arm movements.

#### Laminarity a​nd trapping time

All ANOVAs conducted on %LAM and TT showed significant main effects of group (*p* < 0.001) for all measures, except one (i.e., %LAM ML). Further, the main effect of threat was significant for %LAM AP (*p* < 0.001) and TT AP (*p* = 0.002). The main effect of arm did not reach significance. In addition, there was a group × threat interaction for TT AP (*p* = 0.025, $\:{\eta\:}_{p}^{2} = 0.11$). *Post-hoc* analysis revealed a significant decrease from standing at to above ground (irrespective of arm movement) in young adults (*t* = 3.828, *p* = 0.002, *d* = 0.80) but not in children (*t* = 0.639, *p* = 1.000, *d* = 0.13).

Sensitivity analyses showed that the threat x arm interaction for ENT ML and LINE ML were significant under all parameter settings except the delay based on the average mutual information, whereas the interaction for %DET ML was significant in only three of seven alternative settings. Further, the group x threat interaction for TT AP was significant under all parameter settings except the delay based on average mutual information (Table S 3, supplementary material). The two-dimensional analysis showed main effects of group (*p* < 0.001) and threat (*p* ≤ 0.029; LINE: *p* = .52) as well as a threat x arm interaction for %LAM only (*p* = 0.039) but no group x threat interactions (*p* ≥ 0.164) (Table S4, supplementary material).

#### Associations between linear (COP metrics) and non-linear (RQA metrics) measures

RQA parameters were positively associated with RMS (r_rm = 0.26 to 0.91) and negatively with sample entropy (r_rm = − 0.26 to − 0.55) of the same sway direction (Figure S1, supplementary material).

**Table 3:** Group mean values ± standard deviations for all assessed variables by age group (children vs. young adults), threat conditions (no threat vs. threat), and arm movement strategies (free vs. restricted)

Parameter	Arm condition	Children (*n* = 25)		Young adults (*n* = 23)	
		No threat	Threat	No threat	Threat
REC AP (%)	Free	4.95 ± 0.20	4.79 ± 1.072	4.88 ± 0.23	4.38 ± 0.89
	Restricted	5.04 ± 0.97	5.01 ± 0.97	5.05 ± 1.00	4.71 ± 0.93
REC ML (%)	Free	4.97 ± 0.26	4.35 ± 1.48	5.10 ± 0.27	4.69 ± 1.26
	Restricted	5.14 ± 2.15	5.83 ± 3.58	5.06 ± 1.42	5.51 ± 4.74
DET AP (%)	Free	99.79 ± 0.05	99.75 ± 0.08	99.88 ± 0.02	99.86 ± 0.03
	Restricted	99.78 ± 0.05	99.74 ± 0.10	99.87 ± 0.03	99.87 ± 0.03
DET ML (%)	Free	99.74 ± 0.06	99.61 ± 0.18	99.85 ± 0.05	99.83 ± 0.17
	Restricted	99.67 ± 0.20	99.67 ± 0.18	99.85 ± 0.11	99.85 ± 0.12
ENT AP (nat)	Free	3.34 ± 0.08	3.30 ± 0.12	3.65 ± 0.04	3.62 ± 0.07
	Restricted	3.33 ± 0.10	3.30 ± 0.13	3.66 ± 0.06	3.66 ± 0.07
ENT ML (nat)	Free	3.27 ± 0.12	3.14 ± 0.17	3.61 ± 0.04	3.54 ± 0.20
	Restricted	3.23 ± 0.21	3.25 ± 0.22	3.59 ± 0.15	3.58 ± 0.25
LINE AP (points)	Free	13.47 ± 0.96	13.02 ± 1.29	17.80 ± 0.62	17.55 ± 1.00
	Restricted	13.35 ± 1.21	12.96 ± 1.40	18.02 ± 0.89	18.04 ± 1.20
LINE ML (points)	Free	12.66 ± 1.42	11.54 ± 1.31	17.42 ± 1.20	16.38 ± 1.84
	Restricted	12.37 ± 2.0	12.72 ± 2.54	17.14 ± 1.68	17.26 ± 4.77
LAM AP (%)	Free	99.83 ± 0.04	99.79 ± 0.08	99.91 ± 0.02	99.87 ± 0.08
	Restricted	99.82 ± 0.06	99.79 ± 0.09	99.90 ± 0.04	99.89 ± 0.04
LAM ML (%)	Free	99.79 ± 0.06	99.54 ± 0.66	99.89 ± 0.02	99.16 ± 3.49
	Restricted	99.58 ± 0.67	99.71 ± 0.21	98.87 ± 4.94	98.9 ± 4.65
TT AP (points)	Free	11.10 ± 1.17	10.82 ± 1.80	13.16 ± 2.05	11.33 ± 1.73
	Restricted	11.24 ± 1.85	11.03 ± 1.80	13.67 ± 2.61	12.41 ± 2.15
TT ML (points)	Free	11.00 ± 1.84	9.50 ± 1.90	14.14 ± 2.46	12.85 ± 2.74
	Restricted	10.71 ± 2.93	11.42 ± 3.82	13.89 ± 3.01	13.84 ± 8.81

AP, anteroposterior; DET, determinism; ENT, entropy, ML, mediolateral; LAM, laminarity; LINE, line length; nat, natural units of information; REC, recurrence rate (retained as a descriptive check but not as a primary inferential outcome); TT, trapping time

**Table 4:** Main and interaction effects of the repeated measures ANOVA for all assessed variables

Parameter	Group (children vs. young adults)	Threat (no threat vs. threat)	Arm (free vs. restricted)	Threat × Arm	Group × Threat	Group × Arm	Group × Threat × Arm
DET AP (%)	***F*** **= 93.273** ***p (η***_**p**_^**2**^ **) < 0.001 (0.67)**	***F*** **= 8.832** ***p (η***_**p**_^**2**^ **) = 0.005 (0.16)**	*F* = 0.449 *p (η*_p_^2^) = 0.506 (0.01)	*F* = 0.441 *p (η*_p_^2^) = 0.510 (0.01)	*F* = 1.942 *p (η*_p_^2^) = 0.170 (0.04)	*F* = 1.137 *p (η*_p_^2^) = 0.292 (0.02)	*F* = 0.492 *p (η*_p_^2^) = 0.487 (0.01)
DET ML (%)	***F*** **= 28.285** ***p (η***_**p**_^**2**^ **) < 0.001 (0.38)**	***F*** **= 7.043** ***p (η***_**p**_^**2**^ **) = 0.011 (0.13)**	*F* = 0.026 *p (η*_p_^2^) = 0.873 (0.00)	***F*** **= 4.101** ***p (η***_**p**_^**2**^ **) = 0.049 (0.08)**	*F* = 3.405 *p (η*_p_^2^) = 0.071 (0.07)	*F* = 0.374 *p (η*_p_^2^) = 0.544 (0.01)	*F* = 2.065 *p (η*_p_^2^) = 0.157 (0.04)
ENT AP (nat)	***F =*** **318.649** ***p (η***_**p**_^**2**^ **) < 0.001 (0.87)**	***F*** **= 5.128** ***p (η***_**p**_^**2**^ **) = 0.028 (0.10)**	*F* = 0.755 *p (η*_p_^2^) = 0.389 (0.02)	*F* = 0.465 *p (η*_p_^2^) = 0.499 (0.01)	*F* = 1.132 *p (η*_p_^2^) = 0.293 (0.02)	*F* = 2.887 *p (η*_p_^2^) = 0.096 (0.06)	*F* = 0.189 *p (η*_p_^2^) = 0.666 (0.00)
ENT ML (nat)	***F =*** **103.774** ***p (η***_**p**_^**2**^ **) < 0.001 (0.69)**	***F*** **= 4.675** ***p (η***_**p**_^**2**^ **) = 0.036 (0.09)**	*F* = 1.038 *p (η*_p_^2^) = 0.314 (0.02)	***F*** **= 5.427** ***p (η***_**p**_^**2**^ **) = 0.024 (0.11)**	*F* = 0.028 *p (η*_p_^2^) = 0.869 (0.00)	*F* = 0.139 *p (η*_p_^2^) = 0.711 (0.00)	*F* = 0.938 *p (η*_p_^2^) = 0.338 (0.02)
LINE AP (points)	***F*** **= 406.062** ***p (η***_**p**_^**2**^**) < 0.001 (0.90**)	*F* = 3.249 *p (η*_p_^2^) = 0.078 (0.07)	*F* = 1.373 *p (η*_p_^2^) = 0.247 (0.03)	*F* = 0.475 *p (η*_p_^2^) = 0.494 (0.01)	*F* = 1.067 *p (η*_p_^2^) = 0.307 (0.02)	*F* = 3.628 *p (η*_p_^2^) = 0.063 (0.07)	*F* = 0.178 *p (η*_p_^2^) = 0.674 (0.00)
LINE ML (points)	***F*** **= 153.050** ***p (η***_**p**_^**2**^**) < 0.001 (0.77**)	***F*** **= 1.733** ***p (η***_**p**_^**2**^ **) = 0.195 (0.04)**	*F* = 1.199 *p (η*_p_^2^) = 0.279 (0.03)	***F*** **= 4.586** ***p (η***_**p**_^**2**^ **) = 0.038 (0.09)**	*F* = 0.015 *p (η*_p_^2^) = 0.904 (0.00)	*F* = 0.044 *p (η*_p_^2^) = 0.835 (0.00)	*F* = 0.069 *p (η*_p_^2^) = 0.794 (0.00)
LAM AP (%)	***F =*** **59.721** ***p (η***_**p**_^**2**^ **) < 0.001 (0.57)**	***F*** **= 14.224** ***p (η***_**p**_^**2**^ **) < 0.001 (0.24)**	*F* = 0.076 *p (η*_p_^2^) = 0.783 (0.00)	*F* = 1.753 *p (η*_p_^2^) = 0.192 (0.04)	*F* = 0.096 *p (η*_p_^2^) = 0.758 (0.00)	*F* = 1.450 *p (η*_p_^2^) = 0.235 (0.03)	*F* = 0.191 *p (η*_p_^2^) = 0.664 (0.00)
LAM ML (%)	*F=*0.457 *p (η*_p_^2^) = 0.503 (0.01)	*F* = 1.617 *p (η*_p_^2^) = 0.210 (0.03)	*F* = 1.136 *p (η*_p_^2^) = 0.292 (0.02)	*F* = 2.161 *p (η*_p_^2^) = 0.148 (0.05)	*F* = 0.783 *p (η*_p_^2^) = 0.381 (0.02)	*F* = 1.000 *p (η*_p_^2^) = 0.323 (0.02)	*F* = 0.254 *p (η*_p_^2^) = 0.617 (0.01)
TT AP (points)	***F*** **= 28.259** ***p (η***_**p**_^**2**^**) < 0.001 (0.38**)	***F*** **= 10.276** ***p (η***_**p**_^**2**^ **) = 0.002 (0.18)**	*F* = 3.586 *p (η*_p_^2^) = 0.065 (0.07)	*F* = 0.336 *p (η*_p_^2^) = 0.565 (0.01)	***F*** **= 5.384** ***p (η***_**p**_^**2**^ **) = 0.025 (0.11)**	*F* = 1.429 *p (η*_p_^2^) = 0.238 (0.03)	*F* = 0.211 *p (η*_p_^2^) = 0.648 (0.01)
TT ML (points)	***F*** **= 23.882** ***p (η***_**p**_^**2**^**) < 0.001 (0.34**)	*F* = 0.859 *p (η*_p_^2^) = 0.359 (0.02)	*F* = 1.029 *p (η*_p_^2^) = 0.316 (0.02)	*F* = 2.662 *p (η*_p_^2^) = 0.110 (0.06)	*F* = 0.057 *p (η*_p_^2^) = 0.813 (0.00)	*F* = 0.140 *p (η*_p_^2^) = 0.710 (0.00)	*F* = 0.212 *p (η*_p_^2^) = 0.647 (0.01)

0.02 ≤ *η*_p_^2^ ≤ 0.12 indicates small, 0.13 ≤ *η*_p_^2^ ≤ 0.25 indicates medium, and *η*_p_^2^ ≥ 0.26 indicates large effects. Bold values indicate statistically significant differences (*p* < 0.05). AP, anteroposterior; DET, determinism; ENT, entropy; ML, mediolateral; LAM, laminarity; LINE, line length; nat, natural units of information; TT, trapping time

### Discussion

As assumed in the first hypothesis, the main effect of threat was again significant and revealed decreases for nearly all RQA parameters when standing at height compared to ground. Thus, even for the potentially more demanding semi-static task, reduced predictability and decreased complexity of postural control, more random and less discontinuous COP movements, and shorter durations of CoP fluctuations were observed because of height-induced postural threat, which adds value compared with the previously published linear analyses (Hill et al. 2024; Wissmann et al. 2026). Wissmann and colleagues (2026) also detected significant changes in COP frequency when standing above compared to on ground level (Table S5, supplementary material).

Although significant threat-by-arm interactions were observed, as predicted in the second hypothesis and shown by using linear analyses (Wissmann et al. 2026) (Table S5, supplementary material), they were in the opposite direction. More specifically, *post-hoc* analyses showed decreases in RQA parameters (i.e., %DET ML, ENT ML, and LINE ML) when standing above ground with free but not with restricted arm movements. In other words, free but not (as expected) restricted arms led to reduced predictability of postural control, decreased complexity, and lower stability of predictable movement patterns. However, these interactions were only partly robust to the choice of the RQA parameters (Table S3, supplementary material), and the following interpretation should therefore be regarded as tentative. We propose that these unexpected balance responses are likely to reflect a strategy adapted to the specific requirements of the task. Standing on one leg while performing a continuous contralateral leg swing resulted in a greater number of degrees of freedom compared to the quiet tandem stance used in Experiment 1. Allowing additional free arm movements further increases the degrees of freedom that need to be controlled. Consequently, predictability, complexity, and stability decreased. Conversely, the degrees of freedom to be controlled were lower while standing at height with restricted arms, thus predictability, complexity, and stability remained unchanged.

In agreement with our third hypothesis and previous research using linear analysis methods (Wissmann et al. 2026) (Table S5, supplementary material), the detection of a significant group-by-threat interaction again indicates discrepancies in postural control between children and young adults. Precisely, *post-hoc* analyses revealed a decrease in TT AP (i.e., how long the system remains in a stable state) from ground to height in young adults but not in children. This interaction did not reach significance when the time delay was based on the average mutual information (*p* = 0.059, Table S5, supplementary material).

Thus, young adults but not children adapted their balance control to height-induced postural threat. This finding suggests that young adults, in contrast to children, are capable of appropriately adapting their postural control when confronted with balance-threatening situations such as standing at height. The lack of such an adaptation in children may reflect an immature postural control strategy due to the ongoing maturation of the underlying neural and sensorimotor mechanisms (Shumway-Cook & Woollacott, 1985).

## General discussion

Using RQA, the aim of both experiments was to investigate the effects of height-induced postural threat and restriction of arm movements on postural control in children and young using two different balance tasks: quiet tandem stance (Experiment 1) and unipedal stance with contralateral leg swing (Experiment 2). Taken together, the results of both experiments revealed three novel main findings. First, height-induced postural threat led to decreases in RQA parameters. These changes in the temporal dynamics of postural control offer added value compared with conventional linear analyses (COP metrics) and indicate more variable, less predictable, less complex, more random, less discontinuous, and shorter periods of invariant movement patterns. Second, restriction of arm movements while standing at height did not amplify the threat-related reductions in RQA parameters. Third, evidence for threat-related changes was more consistent in young adults than in children, particularly for the measures showing significant group × threat interactions.

### Height-induced postural threat resulted in postural adaptations

One of the purposes of the present experiments was to determine changes in RQA metrics related to height-induced postural threat. Consistent with our hypothesis and previous research (Weinberg et al. 2025), standing at height led to decreases in RQA parameters. Specifically, standing at that height resulted in a protective postural adaptation strategy consisting of a postural sway pattern that was more irregular, less deterministic, less automatic, less similar, less interrupted, more random, and showed shorter periods of CoP fluctuations. In other words, Standing at height resulted in a decrease in predictability and an increase in randomness in the control of posture, which may reflect more frequent movement adjustments, which is related to an increased attentional demand (Zaback et al. 2019). This may be consistent with increased conscious involvement in postural control, as reported in earlier studies (Hill et al. 2023, 2024) on height-induced postural threat, such as increased perceived instability, fear of falling, and conscious motor processing, although this was not measured directly in the present study. While greater conscious control when standing at height is effective (in preventing a loss of balance or a fall), it is also inefficient, as it reduces automaticity of postural control processes (Weinberg et al. 2025).

### Threat-related changes were not amplified by restricted arms

Another objective of the present experiments was to investigate the effect of restricted arm movements while standing at height. Contrary to our assumption, restriction of arm movements while standing at height did not result in any significant reductions in the RQA parameters, whether a static (Experiment 1) or a semi-static (Experiment 2) balance task was used. One possible explanation is that the effect of height-induced postural threat was too pronounced and masked the effect of restricted arm movements. Alternatively, the effect of arm movement restriction was too weak to amplify the observed decreases in the RQA parameters when standing at height. A static balance task (i.e., quiet tandem stance) was used in Experiment 1 and a potentially more dynamically demanding semi-static balance task (i.e., unipedal stance with contralateral leg swing) was applied in Experiment 2. Consequently, restriction of arm movements should have a greater effect in Experiment 2 than in Experiment 1. In fact, Experiment 2 revealed a threat × arm interaction, but it occurred when participants were standing at height with free rather than restricted arms. Apparently, task-specific factors rather than differences in task demands (static vs. semi-static) account for this finding. As discussed earlier, standing at a height requires more conscious effort to control posture (Hill et al. 2023, 2024). An additional continuous contralateral leg swing while standing increases the number of degrees of freedom that must be controlled, which in turn further increases the effort. Free arm movements allow for more control of the additional degrees of freedom which explains the observed decreases in RQA parameters (i.e., %DET ML, ENT ML, and LINE ML) (Weinberg et al. 2025). It should be noted that the RQA parameters were not derived from present data but chosen for comparability based on a previous study (Weinberg et al. 2025). The delay suggested by the average mutual information was considerably longer, and false nearest neighbors suggested and embedding dimension of four for some groups. Sensitivity analyses showed that the age-related findings of Experiment 1 were robust across all parameter settings examined, whereas several threat-related effects in Experiment 2, including the threat × arm interactions, depending on the time delay. Moreover, %DET and %LAM approached ceiling with a minimum line length of two samples, increasing it to 100 ms resolved the ceiling without changing the findings of Experiment 1. A two-dimensional RQA confirmed the main effects of group and threat, whereas the age-specific response was confirmed to the AP direction.

### Threat led to different postural adaptations in children versus young adults

One of the most interesting findings in both experiments was that young adults but not children adapted their postural control when standing above ground. Precisely, young adults showed significant decreases in the RQA parameters, whereas children did not show any such changes. The diverging behavior in children compared to young adults is consistent with previous studies (Hill et al. 2024; Omorczyk et al. 2021; Wissmann et al. 2025a, 2026) that used traditional linear analysis methods. For children, the authors did not find the typical “stiffening” response (i.e., characterized by decreased sway amplitude and increased sway frequency) that was observed in adults when standing at height versus on ground. The absence of this response in children could be explained by an incomplete maturation of the underlying neural and sensorimotor mechanisms (Haddad et al. 2013; Shumway-Cook and Woollacott 1985; Steindl et al. 2006). In fact, several studies (Hirabayashi & Iwasaki, 1995; Orendorz-Frączkowska and Kubacka 2019; Sinno et al. 2021) using the Sensory Organization Test have shown differences in the processing of sensory inputs for postural control between children and young adults. Their findings suggested that proprioceptive function is the first sensory modality to mature, achieving levels comparable to those of young adults by approximately 5–8 years of age. Visual function reaches adult-like maturity later, around 12–14 years of age, whereas vestibular function demonstrates the slowest developmental course, attaining mature characteristics between 15 and 17 years of age. This pattern was robust across all RQA parameter settings in Experiment 1, whereas in Experiment 2 it was limited to TT AP and less robust.

The observed differences between children and young adults are particularly relevant from a practical perspective, as the failure to utilize adaptive mechanisms in children can lead to an increased risk of falling. Consequently, children should be gradually introduced to elevated standing positions as part of balance assessment and training.

### Non-linear measures complement linear measures

Non-linear measures (RQA metrics) covaried mainly with linear measures (e.g., RMS and sample entropy) of the same sway direction (Figure S1, supplementary material). As the recurrence threshold was scaled to each trial’s standard deviation, this does not reflect a mechanical dependence on sway magnitude but indicates that conditions with larger sway also showed more recurrent and deterministic sway. The association with sample entropy suggests that both approaches partly capture regularity. However, the overlap was incomplete and varied across experiments, age groups and sway directions, indicating that RQA parameters complement rather than duplicate linear measures.

## Conclusions

To our knowledge, these are among the first results showing that RQA can identify age differences in postural control due to height-induced postural threat. Regardless of balance task demands (static or semi-static), young adults showed decreases in the RQA parameters (Experiment 1: %DET AP, ENT AP, %LAM AP; Experiment 2: TT AP) when standing at height, whereas children showed no changes. Restriction of arm movements did not amplify threat-related reductions in RQA parameters. Accordingly, young adults are able to respond to height-induced postural threat with less predictable and more random postural control. In contrast, children showed less evidence of these threat-related changes, which can most likely be explained by incomplete maturation processes in postural control mechanisms. The observed differences between children and young adults should be considered in testing and training scenarios.

## Supplementary Information

Below is the link to the electronic supplementary material.

Supplementary Material 1

Supplementary Material 2

Supplementary Material 3

Supplementary Material 4

Supplementary Material 5

Supplementary Material 6

Supplementary Material 7

## Data Availability

The original contributions presented in the study are included in the article/Supplementary Material, further inquiries can be directed to the corresponding author.
