# Supplementary material for: Recurrence quantification analysis of the effects of height-induced postural threat and arm movement restriction on balance performance in children and young adults
Source: Exp Brain Res. 2026 Nov 8;245(1):2. doi: 10.1007/s00221-026-07428-1 (PMC13745397; doi:10.1007/s00221-026-07428-1)
Supplement: Supplementary file 1 [file 221_2026_7428_MOESM5_ESM.docx]

**Table S1.** Input parameter (radius) used in the recurrence quantification analysis for each participant during tandem stance.

| ***n*** | **Children** | | **Young adults** | |
| --- | --- | --- | --- | --- |
|  | AP | ML | AP | ML |
| 1 | 0.258 | 0.211 | 0.196 | 0.258 |
| 2 | 0.258 | 0.227 | 0.258 | 0.258 |
| 3 | 0.227 | 0.180 | 0.087 | 0.227 |
| 4 | 0.196 | 0.227 | 0.103 | 0.258 |
| 5 | 0.180 | 0.227 | 0.134 | 0.288 |
| 6 | 0.227 | 0.288 | 0.149 | 0.319 |
| 7 | 0.165 | 0.227 | 0.258 | 0.350 |
| 8 | 0.134 | 0.288 | 0.227 | 0.288 |
| 9 | 0.242 | 0.288 | 0.258 | 0.381 |
| 10 | 0.196 | 0.258 | 0.350 | 0.319 |
| 11 | 0.227 | 0.196 | 0.319 | 0.288 |
| 12 | 0.196 | 0.258 | 0.134 | 0.319 |
| 13 | 0.227 | 0.227 | 0.227 | 0.319 |
| 14 | 0.258 | 0.242 | 0.165 | 0.288 |
| 15 | 0.196 | 0.258 | 0.227 | 0.288 |
| 16 | 0.196 | 0.227 | 0.165 | 0.381 |
| 17 | 0.227 | 0.288 | 0.196 | 0.381 |
| 18 | 0.227 | 0.227 | 0.350 | 0.288 |
| 19 | 0.227 | 0.211 | 0.258 | 0.350 |
| 20 | 0.149 | 0.211 | 0.227 | 0.381 |
| 21 | 0.180 | 0.227 | 0.165 | 0.381 |
| 22 | 0.227 | 0.227 | 0.258 | 0.350 |
| 23 | 0.227 | 0.288 | 0.288 | 0.350 |
| 24 | 0.134 | 0.258 |  |  |
| 25 | 0.211 | 0.258 |  |  |
| 26 | 0.134 | 0.227 |  |  |

*Note.* AP, anteroposterior; ML, mediolateral; *n*, participant number
