# Supplementary material for: Recurrence quantification analysis of the effects of height-induced postural threat and arm movement restriction on balance performance in children and young adults
Source: Exp Brain Res. 2026 Nov 8;245(1):2. doi: 10.1007/s00221-026-07428-1 (PMC13745397; doi:10.1007/s00221-026-07428-1)
Supplement: Supplementary file 2 [file 221_2026_7428_MOESM6_ESM.docx]

**Table S5.** Systematic comparison between the previous findings (Wissmann et al. 2025a, 2026) using linear measures (COP metrics) and the present results using non-linear measures (RQA metrics)

|  | **Experiment 1** |  | **Experiment 2** |  |
| --- | --- | --- | --- | --- |
| **Effect** | **Linear measures** | **Non-linear measures** | **Linear measures** | **Non-linear measures** |
| *Main effects* |  |  |  |  |
| Group  (children *vs.* young adults) | sign. for COP MPF AP, COP MPF ML, COP VEL AP, and COP VEL ML | sign. for all measures | sign. for COP RMS ML, COP MPF AP, and COP MPF ML | sign. for all but measures except LAM ML |
| Threat  (no threat *vs.* threat) | sign. for COP MPF AP, COP MPF ML, COP VEL AP, and COP VEL ML | sign. for all measures | sign. for COP MPF AP and COP MPF ML | sign. for all but measures except LINE AP, LAM ML, and TT ML |
| Arm  (free *vs.* restricted) | sign. for COP RMS AP, COP RMS ML, COP VEL AP, and COP VEL ML | non-sign. for all measures | non-sign. for all measures | non-sign. for all measures |
| *Interaction effects* |  |  |  |  |
| Threat × Arm | sign. for COP RMS ML, and COP VEL ML | non-sign. for all measures | sign. for COP RMS ML | sign. for DET ML, ENT ML, and LINE ML |
| Group × Threat | sign. for COP MPF AP | sign. for DET AP, ENT AP, and LAM AP | non-sign. for all measures | sign. for TT AP |
| Group × Arm | non-sign. for all measures | non-sign. for all measures | non-sign. for all measures | non-sign. for all measures |
| Group × Threat × Arm | non-sign. for all measures | non-sign. for all measures | sign. for COP MPF AP | non-sign. for all measures |

*Note:* AP, anteroposterior; COP, centre of pressure; DET, determinism; ENT, entropy; LAM, laminarity; LINE, line length; nat, natural units of information; ML, mediolateral; MPF, mean power frequency; RMS, root mean square; RQA, recurrence quantification analysis; TT, trapping time; VEL, velocity
